# Molecular Dynamics Simulations Study of the Interactions between Human Dipeptidyl-Peptidase III and Two Substrates

**DOI:** 10.3390/molecules26216492

**Published:** 2021-10-27

**Authors:** Shitao Zhang, Shuai Lv, Xueqi Fu, Lu Han, Weiwei Han, Wannan Li

**Affiliations:** Key Laboratory for Molecular Enzymology and Engineering of Ministry of Education, National Engineering Laboratory for AIDS Vaccine, School of Life Science, Jilin University, Changchun 130012, China; zhangst17@mails.jlu.edu.cn (S.Z.); shuailv20@mails.jlu.edu.cn (S.L.); fxq@mails.jlu.edu.cn (X.F.); luhan@jlu.edu.cn (L.H.); weiweihan@jlu.edu.cn (W.H.)

**Keywords:** human dipeptidyl-peptidase III, substrate, molecular dynamics simulations, conformational changes

## Abstract

Human dipeptidyl-peptidase III (hDPP III) is capable of specifically cleaving dipeptides from the N-terminal of small peptides with biological activity such as angiotensin II (Ang II, DRVYIHPF), and participates in blood pressure regulation, pain modulation, and the development of cancers in human biological activities. In this study, 500 ns molecular dynamics simulations were performed on free-hDPP III (PDB code: 5E33), hDPP III-Ang II (PDB code: 5E2Q), and hDPP III-IVYPW (PDB code: 5E3C) to explore how these two peptides affect the catalytic efficiency of enzymes in terms of the binding mode and the conformational changes. Our results indicate that in the case of the hDPP III-Ang II complex, subsite S1 became small and hydrophobic, which might be propitious for the nucleophile to attack the substrate. The structures of the most stable conformations of the three systems revealed that Arg421-Lys423 could form an α-helix with the presence of Ang II, but only part of the α-helix was produced in hDPP III-IVYPW. As the hinge structure in hDPP III, the conformational changes that took place in the Arg421-Lys423 residue could lead to the changes in the shape and space of the catalytic subsites, which might allow water to function as a nucleophile to attack the substrate. Our results may provide new clues to enable the design of new inhibitors for hDPP III in the future.

## 1. Introduction

Dipeptidyl-peptidase III (DPP III, EC 3.4.14.4) is a zinc metallopeptidase of the M49 family [1,2,3,4] which participates in the metabolic process in human bodies by cleaving dipeptide from the N-terminal of biologically active oligopeptides such as angiotensin II (Ang II) [4,5,6]. The M49 family (MEROPS, the Peptidase Database) [7] of the DPP III family is defined by five conserved amino acid sequence regions, including the unique hexapeptide zinc-binding motif (HEXXGH).

Experimental results have shown that DPP III is involved in many pathophysiological processes, including nociception [8,9], blood pressure regulation [10], and cancer cell defense against oxidative stress [11,12]. A possible role of DPP III in the regulation of oxidative stress has been found through its influence on the Nrf2/KEAP1 signaling pathway, and the mechanism of the binding of human DPP III (hDPP III) to Keap1 has been elucidated [13,14]. Moreover, hDPP III has become a potential marker for the prognosis and treatment of multiple types of cancers.

Until now, the three-dimensional structures of the DPP III enzyme from two eukaryotes, human and yeast, have been unveiled [1,4,15,16,17,18]. The crystal structures of ligand-free human and yeast enzymes revealed an elongated protein molecule with two domains separated by a wide cleft, as well as a very similar overall fold [17].

**Figure 1:**
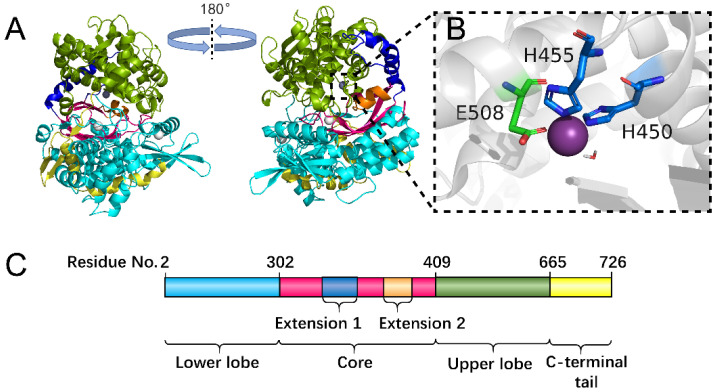
Overall structure of hDPP III. (**A**) Cartoon display of the hDPP III structure with different colors used in different domains. The lower lobe (residue number 2 to 302) is colored in cyan. The β-barrel catalytical core (residue number 303 to 409) is shown in hot pink. There are two α-helix extensions in the catalytical core (Extension 1: residue number 336 to 371; Extension 2: residue number 393 to 404) colored in blue and orange, respectively. The upper lobe with the Zn binding site is shown in green. The C-terminal tail (residue number 666 to 726) is colored in yellow. (**B**) Residues forming the Zn binding site are shown as sticks and the Zn atom is shown as a sphere. (**C**). Linear display of the residue number of each domain of hDPP III.

The division of each domain of the hDPP III protein is shown in Figure 1. From Figure 1A,C, it can be seen that the upper lobe (residue number 410 to 665) is all α-helical and consists of the zinc binding site (Figure 1B). The lower lobe (residue number 2 to 302) exhibits a mixed α- and β-fold with a five-stranded β-barrel forming the catalytical core of hDPP III (Figure 1A,C). Peptides (Ang II and IVYPW) binding to hDPP III are shown in Figure S1 and Figure S2, respectively. From Figures S1 and S2, it can be seen that the peptides primarily interact via their main chain with the catalytical core in the lower lobe.

**Figure 2:**
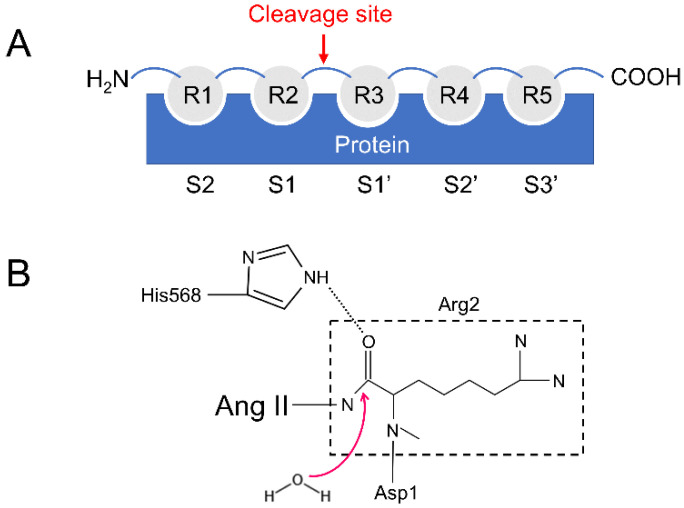
(**A**) The number of each subsite of hDPP III. (**B**) Possible catalytic mechanism of hDPP III.

Gruber and his colleagues have pointed out that the binding of peptides to DPP III is due to entropic changes and the release of water molecules is mainly responsible for the entropy increase [17] (Figure 2A,B). DPP III can bind to and cleave a variety of peptides of different lengths, ranging from tetra- to decapeptides [17,19].

The substrate specificity of peptidases is mostly determined by the shape and size of the corresponding subsites (Sx) in the binding site (Figure 2A) [17,20]. For example, in DPP IV and DPP VII, subsite S1 is small and hydrophobic, restricting residue 1 in the substrate (R1) to proline, alanine, or glycine [20]. In contrast, subsites in hDPP III are deep and slightly hydrophobic (Table S1), which is consistent with the relaxed specificity shown by DPP III [6]. Therefore, the enzyme cleaves other peptides very efficiently, leading to one question: Is the binding mode different between true substrates and inhibitory peptides?

In this study, 500 ns molecular dynamics (MD) simulations were performed on three systems (Details about free-hDPP III (PDB code: 5E33), hDPP III-Ang II (DRVYIHPF, PDB code: 5E2Q), and hDPP III-IVYPW (“slow” substrate, PDB code: 5E3C) [17] are listed in Table 1), to explore the different binding modes and the conformational changes between true substrates and the inhibitory peptides that bind to hDPP III.

## 2. Results and Discussion

The X-ray structure of hDPP III in a complex with the pentapeptide tynorphin showed that ligand binding was accompanied by a large domain motion and a closure of the inter-domain cleft (Figure 1A) [18]. As can be seen from Figure 1A, the lower lobe exhibits a mixed α- and β-fold with a five-stranded β-barrel forming the structural core of this lobe, connected by a number of quite flexible loop regions. In free-hDPP III, two histidine residues (His450 and His455) and Glu508, which are all part of the conserved sequence motifs (HELLGH and EECRAE) in the M49 family, form the characteristic tetrahedral configuration in metalloenzymes (Figure 1B,C). Glu451 in the HELLGH motif that may act as a general base activating the catalytic water molecule functioned as nucleophilic attacking the peptide bond, similar to other zinc dependent proteases such as thermolysin [21,22].

### 2.1. Stabilization and Hydrophilicity of the Systems

**Figure 3:**
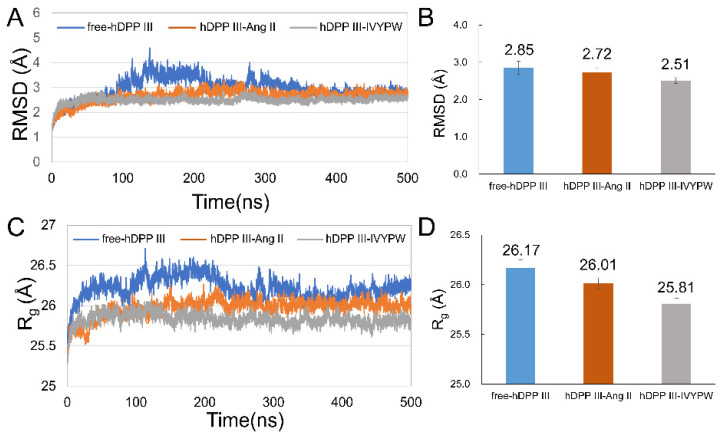
RMSD and R_g_ plots of the three systems throughout 500 ns. (**A**) RMSD plot. (**B**) Average RMSD values of the last 200 ns in the MD simulations. (**C**) R_g_ plot. (**D**) Average R_g_ values of the last 200 ns in the MD simulations.

After 500 ns simulations, the root-mean-square deviations (RMSD) and mean radius of gyration (R_g_) values of the backbone of the three systems were calculated to evaluate the equilibrium of the systems and are shown in Figure 3. The RMSD values of free-hDPP III, hDPP III-Ang II and hDPP III-IVYPW were stabilized at about 2.85 Å, 2.72 Å, and 2.51 Å, respectively (Figure 3B), indicating that the three systems had already reached a state of relative equilibrium. As shown in Figure 3C,D, the R_g_ values of hDPP III-Ang II, and hDPP III-IVYPW were stabilized at 26.01 Å and 25.81 Å, respectively, while the free-hDPP III system was stabilized at 26.17 Å. In this study, three repeated simulations were carried out for each system, and the average structures were generated using the program CPPTRAJ [23] in the AmberTools17 packages by RMS fitting all of the backbone atoms in 5000 snapshots at 0.1 ns intervals and then averaging the coordinates. The RMSD values and the aligned average structures in three repeats are shown in Figure S3.

**Figure 4:**
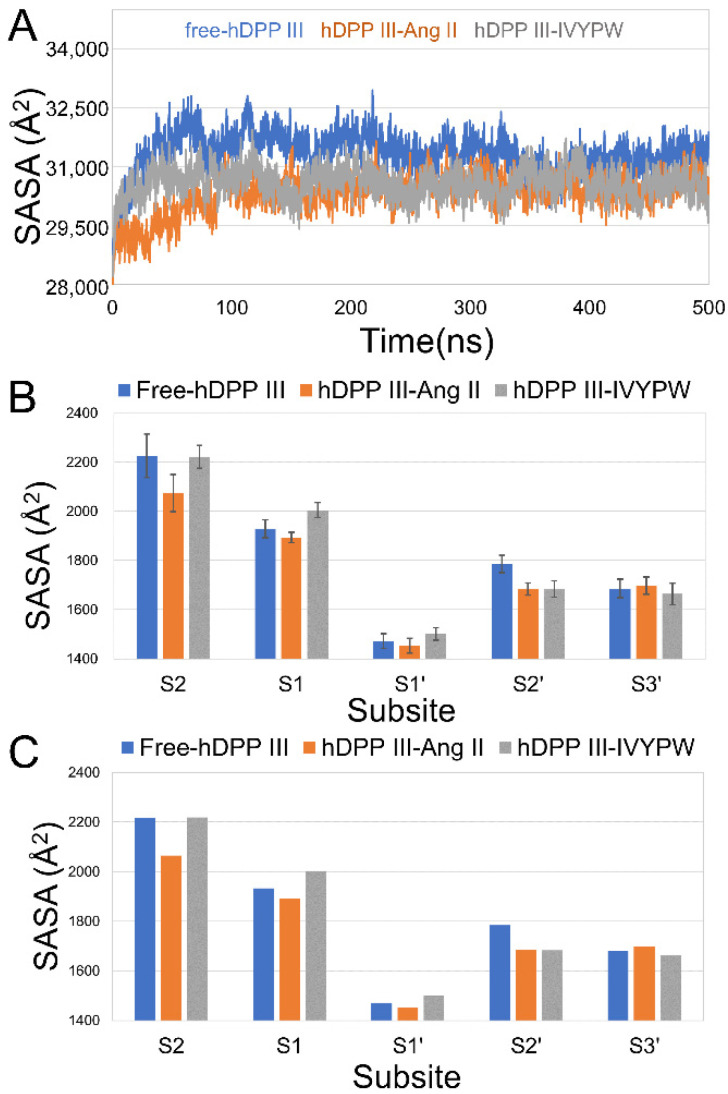
(**A**) SASA plot of the three systems over 500 ns. (**B**) The average of SASA values of each subsite in the last 200 ns. (**C**) The median of the SASA values of each subsite in the last 200 ns.

Subsequently, the hydrophilicity of the three systems was evaluated by solvent accessible surface area (SASA) values throughout the 500 ns MD simulations, and the results are shown in Figure 4. In Figure 4A, it is obvious that the SASA values of free-hDPP III were higher than those of the other two systems in the last 200 ns, which might be due to the relative movement of the upper and lower domains of hDPP III with the influence of the substrates, leading to the closure of the cleave (active site). The average and median of the SASA values of each subsite in the last 200 ns were investigated and are shown in Figure 4B,C, respectively. The SASA values of subsite S1 had lower scores in the hDPP III-Ang II complex than the others, which illustrated the hydrophobic feature restricting R2 for hydrophobic residues (R2 as Arg in Ang II, Val in IVYPW).

### 2.2. The Size of the Catalytic Subsites

**Figure 5:**
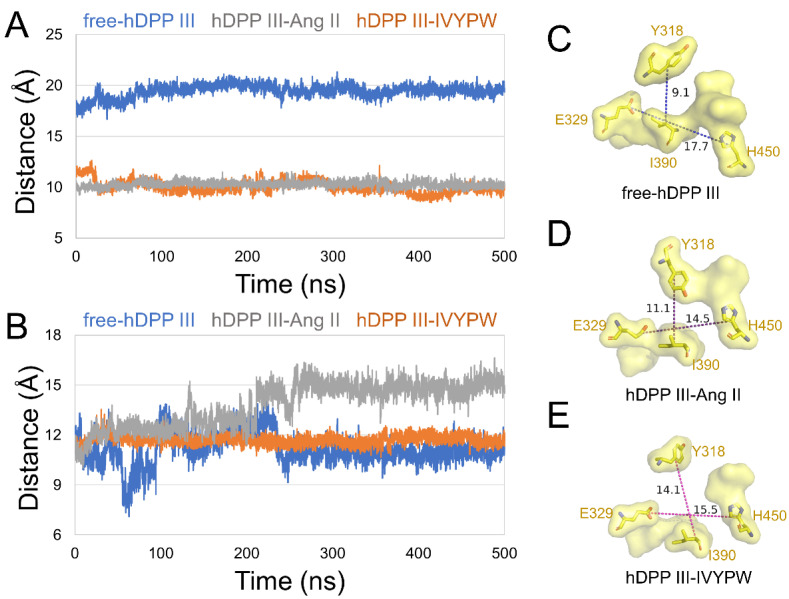
The distance of (**A**) Glu329-His450 and (**B**) Tyr318-Ile390 of subsite S1 in the three systems during 500 ns MD simulations. The surface form displays of subsite S1 in (**C**) free-hDPP III, (**D**) hDPP III-IVYPW, and (**E**) hDPP III-Ang II with residues Tyr318, Glu329, Ile390, and His450 shown as sticks and labeled.

**Figure 6:**
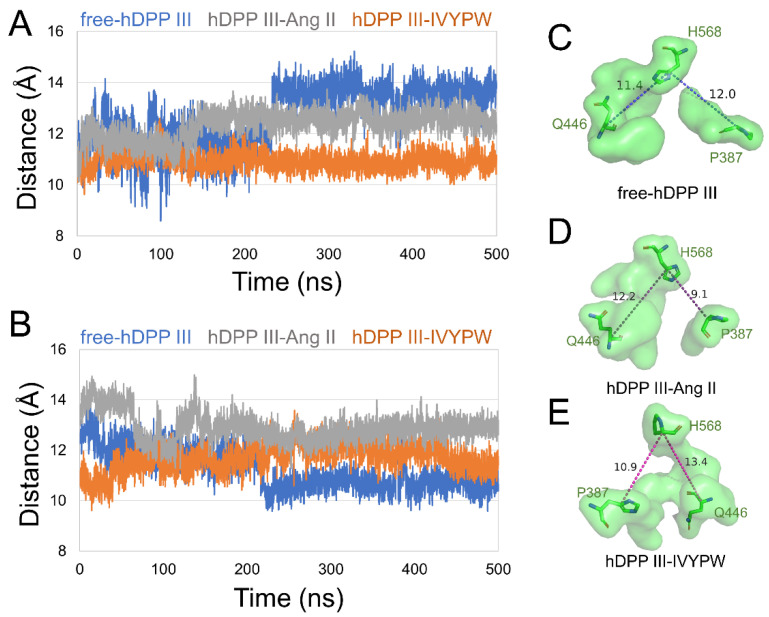
The distance of (**A**) Pro387-His568 (**B**) Gln-His568 of subsite S1′ in the three systems during 500 ns MD simulations. The surface form displays of subsite S1′ in (**C**) free-hDPP III, (**D**) hDPP III-IVYPW, and (**E**) hDPP III-Ang II with residues Pro387, Gln446 and His568 shown as sticks form and labeled.

To unravel the size of subsites S1 and S1′, we characterized the distances between the center of mass in the following residues in subsite S1: (1) Tyr318 and Ile390, (2) Glu329 and His450 (Figure 5), and in subsite S1′: (1) Pro387 and His568, (2) Gln446 and His568 (Figure 6) in the hDPP III. As shown in Figure 5, the distance between the two pairs of residues was enlarged in hDPP III-Ang II, indicating that the volume of subsite S1 in hDPP III-Ang II was expanded during the simulation. In Figure 6, the two groups were at a larger distance from each other in hDPP III-Ang II, which demonstrated that the subsite S1′ in hDPP III-Ang II increased in its size during the 500 ns simulations. The volume of the two subsites remained unchanged in the free protein system. In terms of the hDPP III-Ang II complex, subsite S1 in this composite was large and hydrophobic, which benefited water in the nucleophilic attack on the substrate.

### 2.3. Analysis of Hydrogen Bonds

**Figure 7:**
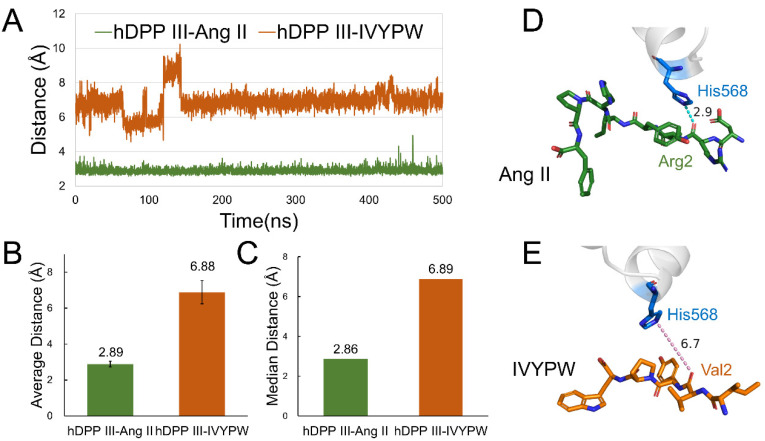
The distance between the ε nitrogen atom in His568 of hDPP III and the oxygen atom in the second residue of the substrates (N-O distance). (**A**) N-O distance throughout the 500 ns molecular dynamics simulations. (**B**) Average N-O distance in the 500 ns simulations. (**C**) Median N-O distance in the 500 ns simulations. (**D**) N-O distance in the hDPP III-Ang II complex. His568 is shown as blue sticks and Ang II is shown as green sticks with Arg2 labeled. (**E**) N-O distance in hDPP III-IVYPW complex. His568 is shown as blue sticks and IVYPW is shown as orange sticks with Val2 labeled.

The probabilities of hydrogen bonds between the two substrates and hDPP III during the 500 ns MD simulations were calculated using the program CPPTRAJ [23] in the AmberTools17 packages and are listed in Table 2. The angle cutoff for the solute–solute hydrogen bonds was 135° and the distance cutoff was set at 4.0 Å. As can be seen from Table 2, the hydrogen bonds were concentrated in subsite S1 and S1′in the hDPP III-Ang II complex. However, in the hDPP III-IVYPW complex, the hydrogen bond formed between the substrate IVYPW and the receptor protein was mainly in subsite S2. It is noteworthy that the hydrogen bond generated by Arg2 in Ang II and His568 in the receptor protein reached a probability of 74.65%. To further explore the hydrogen bond changes in His568, an important catalytic residue, with the substrate during the simulations, the distance between the ε nitrogen atom in His568 of hDPP III and the oxygen atom in the second residue of the substrates (N-O distance) was calculated (Figure 7). As shown in Figure 7, the values of the N-O distance were maintained at about 3 Å in the hDPP III-Ang II complex but exceeded 6 Å in the hDPP III-IVYPW complex. Ang II may interact with His568 through hydrogen bonds to enable it to stabilize the oxyanion in the tetrahedral intermediate as well as in other metalloproteases such as thermolysin.

### 2.4. Flexibility Analysis of hDPP III

**Figure 8:**
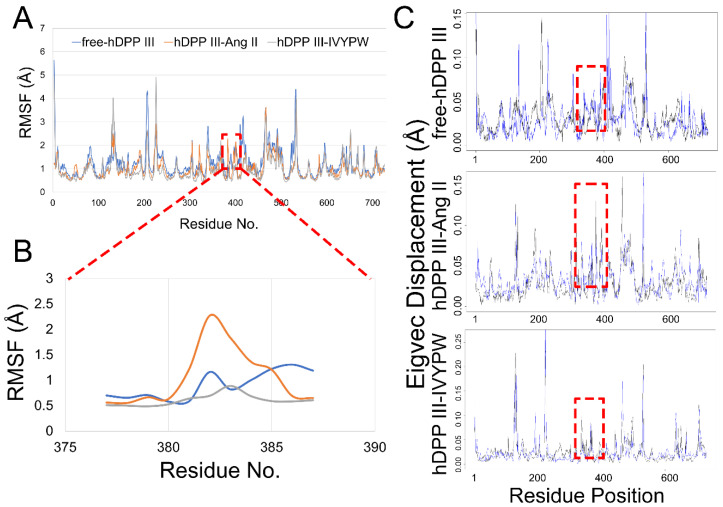
(**A**) RMSF plots of the three systems. (**B**) RMSF plots for the residue Thr380-Gly385 in the three systems. (**C**) Residue displacements that correspond to the motions described by the first eigenvector for free-hDPP III, hDPP III-Ang II, and hDPP III-IVYPW.

**Figure 9:**
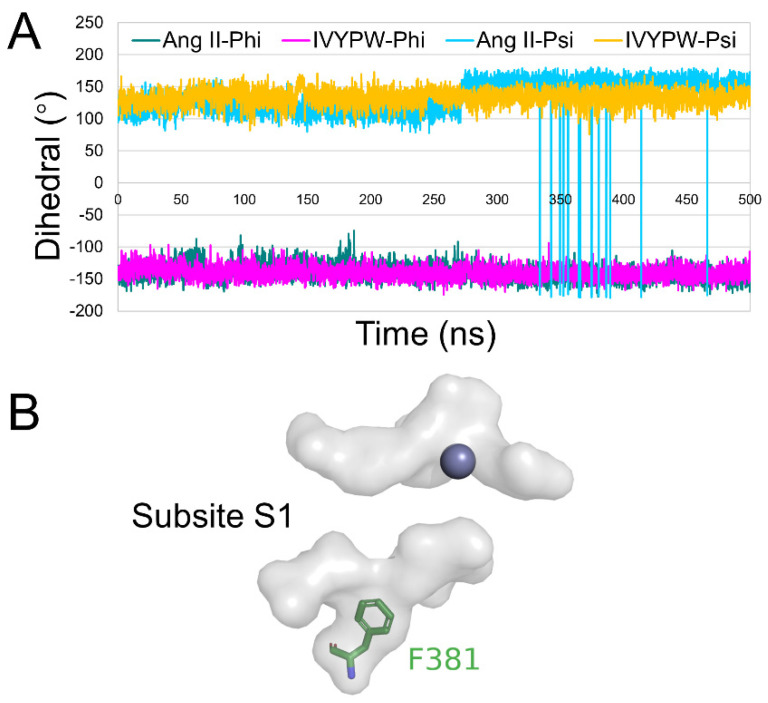
(**A**) Dihedral change of Phe381 in the hDPP III-Ang II and hDPP III-IVYPW complexes during 500 ns simulations. (**B**) A close view of subsite S1 (in the surface form) with Phe381 shown as green sticks and the Zn atom shown as a grey sphere.

The root mean square fluctuation (RMSF) was calculated for the backbone atoms in the three simulations and the residue displacements corresponding to the motions described by the first and second eigenvector for three systems are shown in Figure 8. Residue Phe381-Ser384 exhibited distinct atom-positional fluctuation amplitudes in the hDPP III-Ang II complex and may have been able to change the shape and size of the subsites in the receptor protein. Phe381 is an anchor residue present in subsite S1 (Table S1 and Figure 9). The dihedral angle data show that Phe381 can fluctuate sharply in the presence of Ang II, which may produce a change in the shape of subsite S1. Thus, it is useful for water molecules to carry out a nucleophilic attack on the substrate.

### 2.5. Conformational Changes for Inhibitor Binding

**Figure 10:**
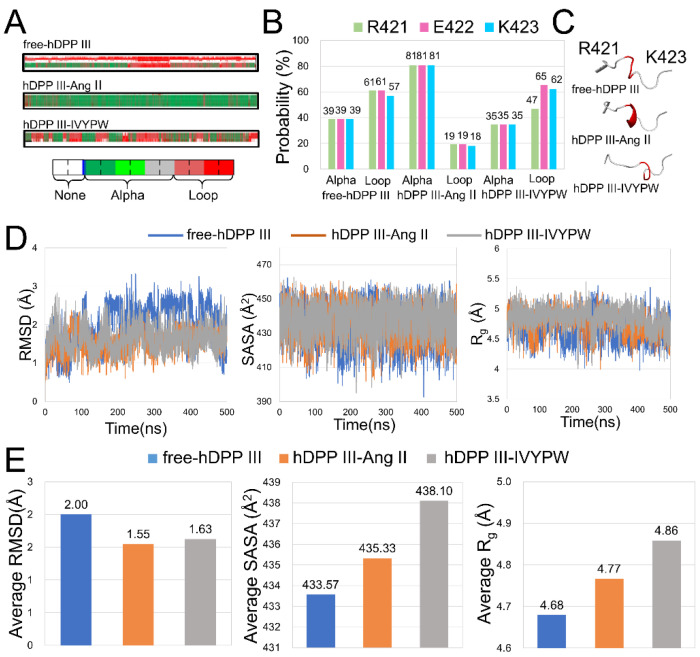
(**A**) DSSP analysis of Arg421-Lys423 residues in three systems throughout 500 ns simulations. (**B**) Probabilities of α-helix and loop for Arg421-Lys423 residues. (**C**) Structures of Arg421-Lys423 residues (red) in cartoon form. (**D**) RMSD, R_g_, and SASA plots for Arg421-Lys423 residues in three systems. (**E**) Average RMSD, R_g_, SASA values for Arg421-Lys423 residues in three systems.

The conformational changes caused by the substrates in the hDPP III-substrate complexes were compared with those in free-hDPP III. In Figure 10A, the Arg421-Lys423 residues formed an α-helix for most of the hDPP III-Ang II simulation process, but only part of the helix was formed in the hDPP III-IVYPW complex and in free-hDPP III. The probabilities of Arg421-Lys423 residues forming an α-helix was calculated (Figure 10B). The α-helix in the Arg421-Lys423 residues had an 81% chance of appearing in hDPP III-Ang II, while the probability in hDPP III-IVYPW was 35%. To facilitate the comparison, the structures of free-hDPP III, hDPP III-Ang II, and hDPP III-IVYPW are shown in Figure 10C. RMSD, R_g_, and SASA values and their averages of the Arg421-Lys423 residues were also calculated (Figure 10D,E). The changes in the three indices indicate that the structure of the Arg421-Lys423 residues in hDPP III-Ang II changed substantially compared with that in free-hDPP III and showed the same trend in three repetitions (Table S2). It was reported that residues 409 to 420 were defined as mechanical hinge residues, where the magnitude of the conformational change in the enzyme caused by entropic costs was involved [17]. Arg421-Lys423 residues could be considered as mechanical hinge residues in the hDPP III protein leading to the movement of the upper and lower lobes of the protein. These conformational changes could cause spatial changes in catalytic sites to promote the decomposition of the substrates.

### 2.6. Principal Component Analysis (PCA) and Free Energy Landscape (FEL) Analysis

**Figure 11:**
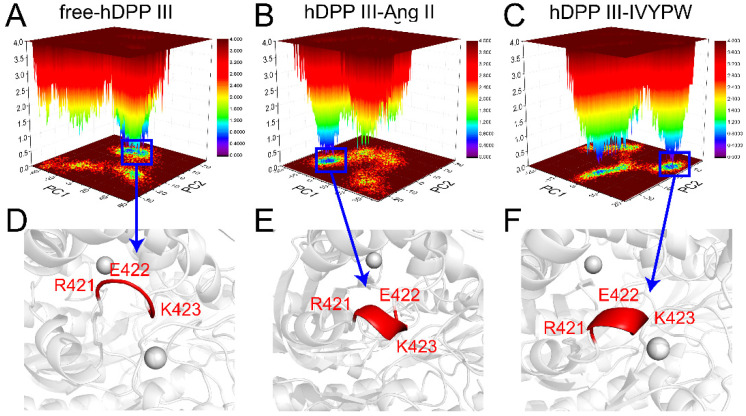
PCA-based FEL analysis of hDPP III. (**A**–**C**) Free-hDPP III, hDPP III-Ang II, and hDPP III-IVYPW as a function of the projections of the MD trajectory onto the first (PC1) and second (PC2) eigenvectors. (**D**–**F**) The structures of the most stable conformations of the three systems are presented with the conformation of Arg421-Lys423 colored in red.

The internal dynamics of the three systems with PCA were investigated and are shown in Figure 11 and Table 3. The structures of the most stable conformations of the three systems revealed that the conformational change in the Arg421-Lys423 residues occurred in hDPP III-Ang II (α-helix generated), while in the hDPP III-IVYPW complex, an α-helix was partly generated. The binding of Ang II could cause conformational changes in hinge residues, thereby leading to a large domain movement upon ligand binding [17] and spatial changes of the active sites, which could promote the decomposition of the substrates.

### 2.7. MM/PBSA and Interaction Energy Results

Binding free energies calculated by MM/PBSA and the interaction energy results are shown in Table 4. The nonbonded van der Waals (E_vdw_), nonbonded electrostatic interactions (E_elec_), electrostatic contribution to the solvation free energy calculated through PB (E_PB_), nonpolar solvation free energy term (E_npolar_), and dispersion contribution (E_disper_) are listed and were calculated to obtain the total binding free energy (ΔTotal). These results indicate that the binding free energy of the hDPP III-Ang II complex (−62.5156 ± 0.78 kcal/mol) was higher than that of the hDPP III-IVYPW complex (−2.03 ± 0.54 kcal/mol). The entropy term of the Ang II composite system (65.99 ± 0.05 kcal/mol) was higher than that of IVYPW (37.59 ± 1.84 kcal/mol) as well. The energy analysis showed that the binding of the Ang II and hDPP III proteins was more effortless and thus the catalytic process was more efficient.

### 2.8. Hotspot Interaction Points Detected by Hierarchical Cluster (HC) Analysis

**Figure 12:**
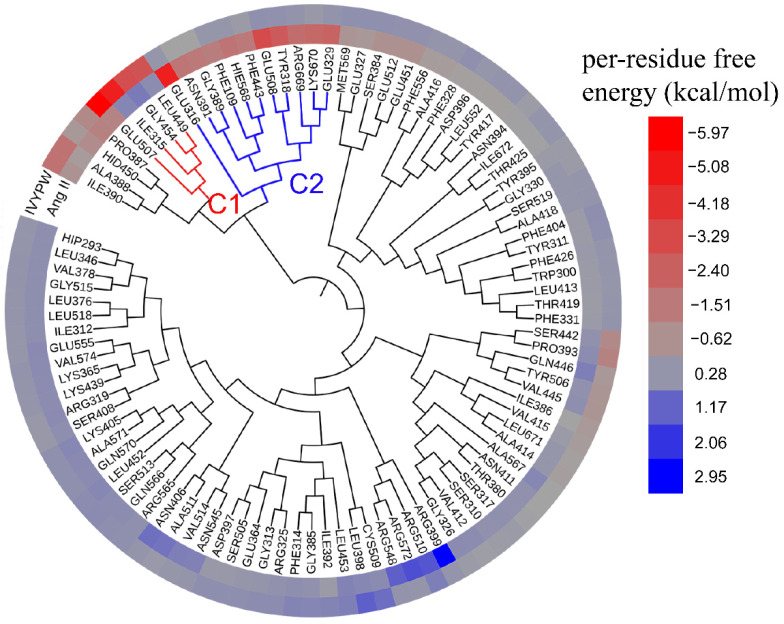
Hierarchical clustering tree with a heatmap of the residues providing contributions to the bindings of substrates to hDPP III based on per-residue energy decomposition. Contributions to binding energy that are beneficial to substrate binding are shown in red and gradually fade towards gray as the binding energy decreases. The binding energy contribution that is not conductive to substrate binging site is shown in blue, and gradually turns to gray as the binding energy decreases.

To analyze the hotspot interaction points and the common binding characteristics of the associations between hDPP III and Ang II and IVYPW, the HC analysis was carried out with the aid of per-residue free energy decomposition (Figure 12). It can be seen from Figure 12 that in the presence of different substrates, the residues that contributed to the free energy of binding were somewhat different.

It can be seen from Figure 12 that in the presence of different substrates, the residues that contributed more to the free energy of binding were assigned into two clusters, C1 (red) and C2 (blue). The residues that mainly contributed to the IVYPW binding on the receptor protein were in cluster C1, including residue Glu507, Ile315, Gly454, and Ieu449, among which only residue Glu507, located at Subsite S2 of hDPP III, functioned catalytically. Residues that contributed favorable energies to the associations of Ang II were Phe109, Tyr318, Glu316, Glu329, Gly389, Asn391, Phe443, Glu508, His568, Arg669, and Lys670 existing in cluster C2. All residues in cluster C2 were located at catalytic subsites of hDPP III and contributed against the binding of IVYPW, indicating that the residues of the catalytic site of hDPP III are more inclined to bind to Ang II.

## 3. Discussion

HDPP III, a zinc-dependent hydrolase that is capable of cleaving dipeptides from the N-terminal of small peptides of various lengths, is involved in many human physiological processes, such as pain perception, blood pressure regulation, and various forms of cancers. In this study, 500 ns MD simulations were performed on free-hDPP III, hDPP III-Ang II, and hDPP III-IVYPW to explore different binding modes and conformational changes among them. The results showed that in the hDPP III-Ang II complex, the subsite S1 became larger and hydrophobic, which made the substrate vulnerable to nucleophilic attack. The most stable conformational structures of the three systems showed that the conformational changes took place in the Arg421-Lys423 residues were relatively large in the hDPP III-Ang II complex. While in the hDPP III-IVYPW complex, the α-helix was only partially generated. As the mechanical hinge in hDPP III, the ordered domain of the Arg421-Lys423 residues facilitates the nucleophilic attack of water molecules on the substrate. The binding of Ang II to hDPP III may lead to changes in the shape and size of subsite S1, an important catalytic site, so as to promote the decomposition of the substrate. The results of this study can provide new clues for the future design of new inhibitors of hDPP III.

## 4. Materials and Methods

### 4.1. MD Simulations

Free-hDPP III (the ligand Met-enkephalin in the crystal structure of PDB code 5E33 was removed and used as an initial structure of the free protein system for further investigation.), hDPP III-Ang II (PDB code: 5E2Q), and hDPP III-IVYPW (PDB code: 5E3C) structures were obtained from the RCSB Protein Data Bank (https://www.rcsb.org, accessed on 13 April 2016) and were used as starting structures. Subsequently, H++ [24] was employed to calculate the pKa of the residues in three systems and to add the missing hydrogen atoms to the crystal structures according to the pH results. Moreover, the hydrogens on the imidazole groups in the histidines were allocated based on the calculated pKa values. Parameters were generated using the ff99SB force field [25]. Systems were solvated in a cubic periodic boundary water box with a side length of 12 Å of TIP3P [26] and sodium ions were randomly added in order to maintain the electrical neutrality of the system. The solvated systems were then minimized through 500 cycles of the steepest descent algorithm and 500 cycles of the conjugate gradient algorithm to achieve the initial equilibrium structure. This was followed by 50 ps of heating up from 0 to 310 K, and 50 ps of density balance. Subsequently, 500 ps of constant pressure balance was performed at 310 K. A 500 ns MD unconstrained simulation was conducted by the Amber16 packages (University of California, San Francisco, CA, USA) [27] with the following conditions strictly defined: (1) electrostatic interactions were calculated using the particle-mesh Ewald (PME) [28] method in each system, (2) bonds involving hydrogen atoms were constrained using the SHAKE algorithm [29], (3) 1 atm constant pressure and 310 K temperature was maintained by the Langevin dynamics method [30]. The total number of steps in each MD simulation was 2.5 × 10^8^ with a time step of 2 × 10^−3^ ps, and the system coordinates were recorded every 0.01 ns. The stability of the system was evaluated by RMSD, RMSF, and R_g_ calculated by VMD [31] TCL scripts.

### 4.2. PCA and FEL Analysis

PCA is a common statistical multivariate method which can select the structure of each frame in a MD trajectory as a new set of variables called principal components (PCs) with minimal loss of information. The starting point of the PCA was the matrix of correlation coefficients derived from the original data set. Firstly, the covariance matrix was constructed from the MD trajectory, and then its eigenvalues and eigen-vectors were calculated. The larger the eigenvalues corresponding to the eigenvectors, the more motion behavior of the system reflected in this eigenvector coordinate. The Bio3D package [32] in R was utilized to examine the relationship between the different conformations sampled during the trajectory. The FEL is used to extract the top two eigenvectors to describe the free energy of the various conformations of the macromolecules.

### 4.3. MM/PBSA Binding Free Energy Calculations, Interaction Entropy Calculations, and Ligand–Residue Energy Decomposition Analysis

MM/PBSA is an analytical technique widely used in MD simulations. The binding free energies of the complexes were calculated by the MMPBSA.py program [33] built into the Amber16 packages through the end-state method using the Poisson–Boltzmann (PB) [34] implicit solvent models. The interaction entropy method [35] was utilized to calculate the entropic contributions to free energies in protein–protein binding systems, in which the MD simulations of two complexes in explicit water were used to generate an ensemble average for the calculation of entropy terms. In addition to global free energies, per-residue decomposition was performed to calculate the contribution of each residue to the total free energy. A total of 1000 frames were extracted from the last 200 ns in each MD simulation for the calculation of the binding free energies.

### 4.4. HC Analysis

The identification of key spots for the interactions between ligands and receptors plays a crucial role in the detection of receptor target sites. Therefore, HC analysis has become an effective technique for investigating the key sites and binding modes in the receptor–ligand complexes [36,37,38,39]. HC analysis in this work was based on the per-residue decomposition results to explore the energy differences between the two substrates towards hDPP III. The analysis was carried out through R packages using Manhattan distance [40] to determine the similarity levels among the energy vectors, and Ward’s minimum variance method to minimize the total variance within the cluster. Finally, the online tree generator iTOL [41] was utilized to conduct the final visualization results.

## Figures and Tables

**Table 1:** Details of the initial structures of the three systems used in this study. (“-” indicates that the ligand of the system was removed for the MD simulations.).

PDB Code	Residue No.	Receptor Length	Ligand	Ligand Length	Ligand Residues
5E33	2-726	725	-	-	-
5E2Q	2-726	725	Ang II	8	DRVYIHPF
5E3C	3-726	724	IVYPW	5	IVYPW

**Table 2:** Probabilities of hydrogen bonds between substrates and hDPP III during 500 ns MD simulations.

Substrate	Subsite	Acceptor	Donor H	Donor	Probability (%)
Ang II	S2	Asp1@O	Asn391@H	Asn391@N	98.97
	S1′	Val3@O	Ala388@H	Ala388@N	97.5
	S1, S1′	Gly389@O	Val3@H	Val3@N	90.39
	S1	Tyr318@O	Arg2@H	Arg2@N	88.05
	S1	Arg2@O	His568@H	His568@N	74.65
	S1, S1′	Val2@O	Gly389@H	Gly389@N	67.18
	S2′	Tyr4@O	Arg572@H	Arg572@N	65.58
	S1	Glu329@O	Arg2@H	Arg2@N1	63.39
	S2′	Tyr4@O	Arg572@H	Arg572@N	61.65
	S1	Glu329@O1	Arg2@H	Arg2@N2	60.54
	S1	Glu329@O2	Arg2@H	Arg2@N3	56.13
IVYPW	S2	Ile1@O	Asn391@H	Asn391@N	98.87
	S1′, S2′	Tyr3@O	Ala388@H	Ala388@N	94.92
	S1, S1′	Gly389@O	Tyr3@H	Tyr3@N	90.09
	S1, S1′	TYR_727@O	Gly389@H	Gly389@N	79.04
	S2	Asn391@O	Ile1@H1	Ile1@N	31.29
	S2	Asn391@O	Ile1@H2	Ile1@N	31.11
	S2	Asn391@O	Ile1@H3	Ile1@N	30.92

**Table 3:** The probabilities of PC1 and PC2 of the three systems.

	PC1 (%)	PC2 (%)
Free-hDPP III	28.33	15.35
hDPP III-Ang II	17.44	14.13
hDPP III-IVYPW	13.74	11.5

**Table 4:** MM/PBSA and interaction entropy results for two enzyme–ligand systems (kcal/mol).

	hDPP III-Ang II	hDPP III-IVYPW
E_vdW_	−88.68 ± 0.34	−67.55 ± 0.22
E_elec_	−553.42 ± 1.43	−261.78 ± 1.67
E_PB_	512.63 ± 1.24	284.67 ± 1.65
E_npolar_	−83.09 ± 0.10	−52.75 ± 0.10
E_disper_	150.05 ± 0.10	95.39 ± 0.10
ΔG_gas_	−642.01 ± 1.39	−329.33 ± 1.66
ΔG_solv_	579.58 ± 1.25	327.31 ± 1.65
ΔTotal	−62.52 ± 0.78	−2.03 ± 0.54
IE	65.99 ± 0.05	37.59 ± 1.84

## Data Availability

Data are contained within the article or Supplementary Material.

## Supplementary Materials

The following are available online, Figure S1: Interactions of hDPP III-Ang II. Ang II is shown in purple lines. Residues of hDPP III is shown as yellow lines. Figure S2: Interactions of hDPP III-IVYPW. IVYPW is shown in purple lines. Residues of hDPP III is shown as yellow lines. Figure S3: RMSD values and the aligned average structures of three systems during 500 ns MD simulations. Figure S4: RMSF plots of three systems. Table S1: Residues contained in each subsite. Table S2: The probability of secondary structures of residue Arg421-Lys423 in three repetitions.

## Sample Availability

Not available.
