# Supplementary material for: Molecular Dynamics Simulations Study of the Interactions between Human Dipeptidyl-Peptidase III and Two Substrates
Source: Molecules. 2021 Oct 27;26(21):6492. doi: 10.3390/molecules26216492 (PMC8587566; doi:10.3390/molecules26216492)
Supplement: Supplementary file 1 [file molecules-26-06492-s001.zip › molecules-1416118-supplementary.pdf]

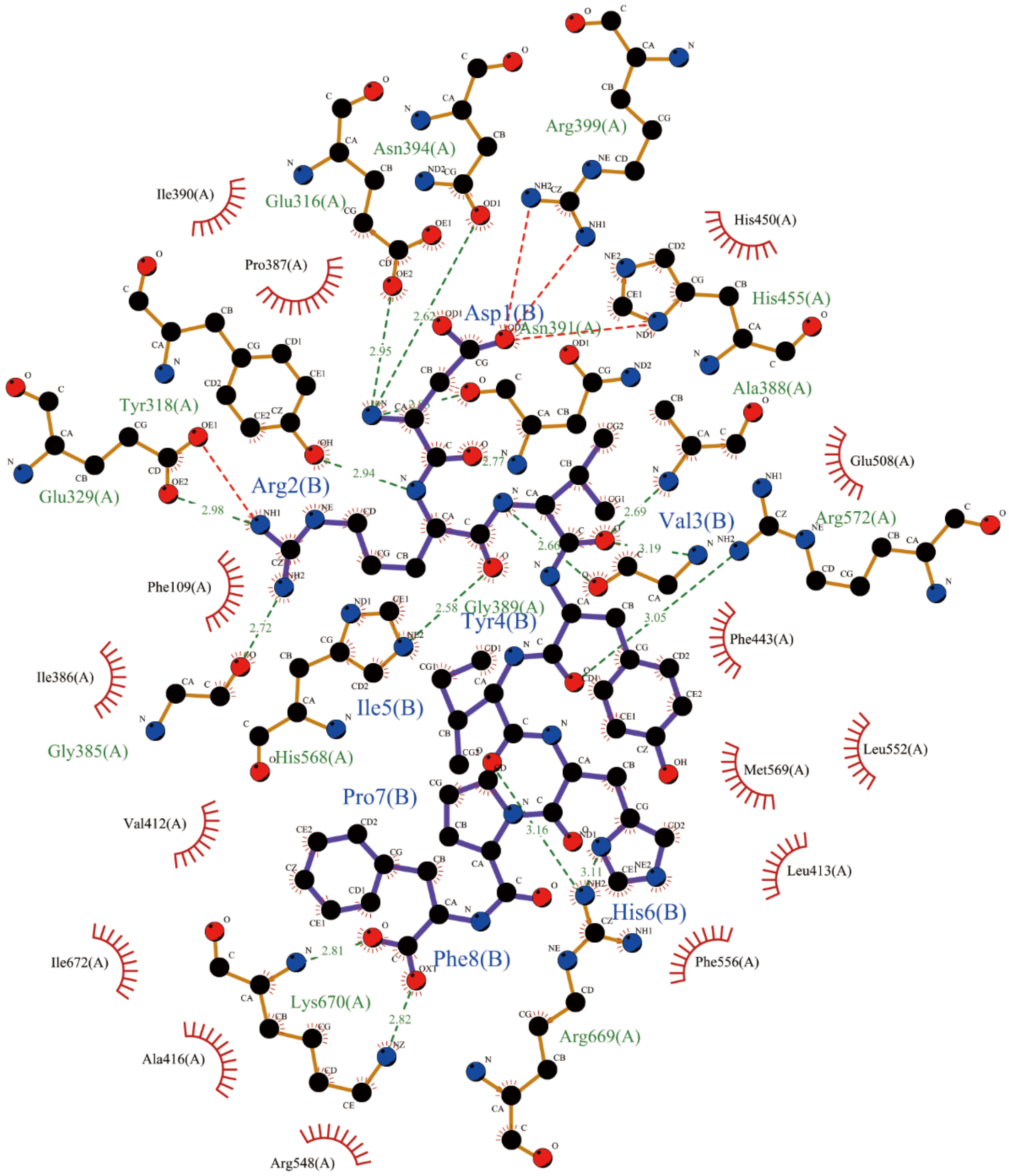

Figure S1. Interactions of hDPP III-Ang II. Ang II is shown in purple lines. Residues of hDPP III is shown as yellow lines.

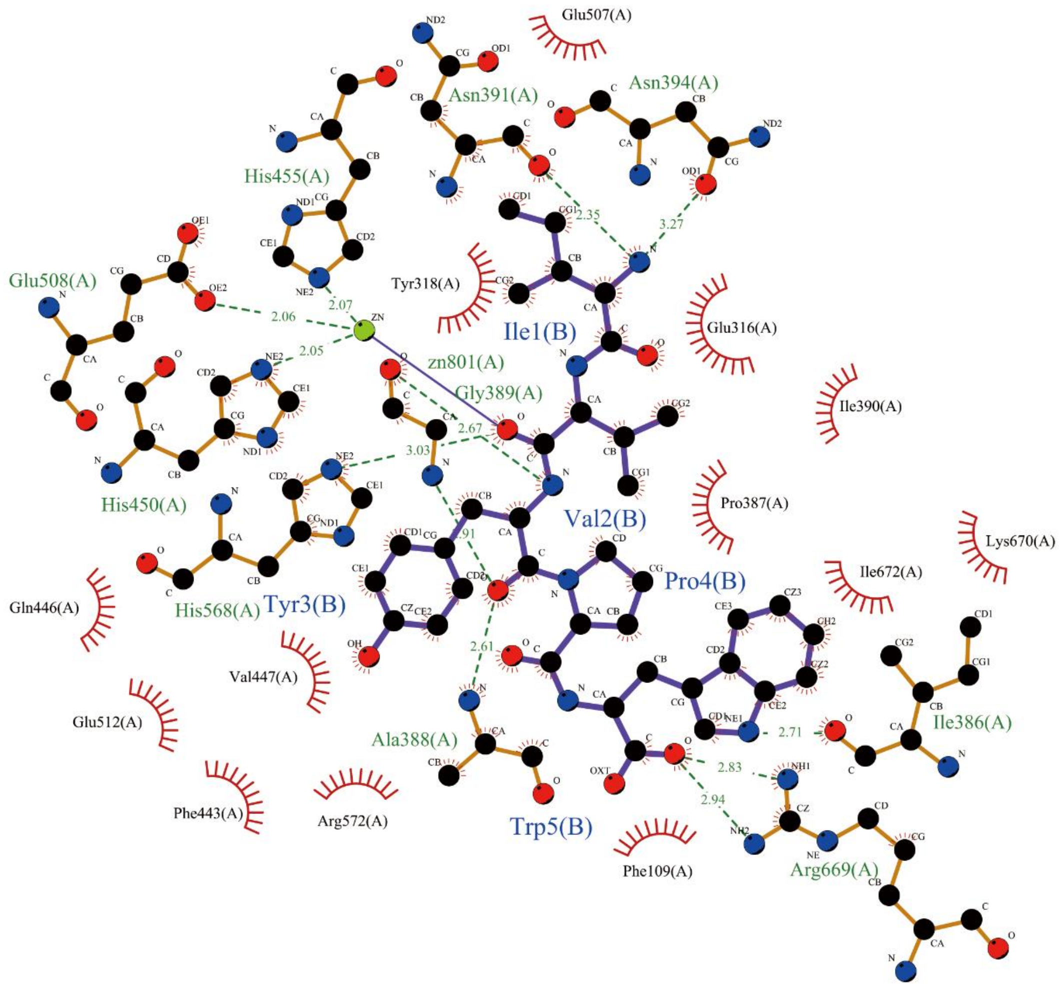

Figure S2. Interactions of hDPP III-IVYPW. IVYPW is shown in purple lines. Residues of hDPP III is shown as yellow lines.

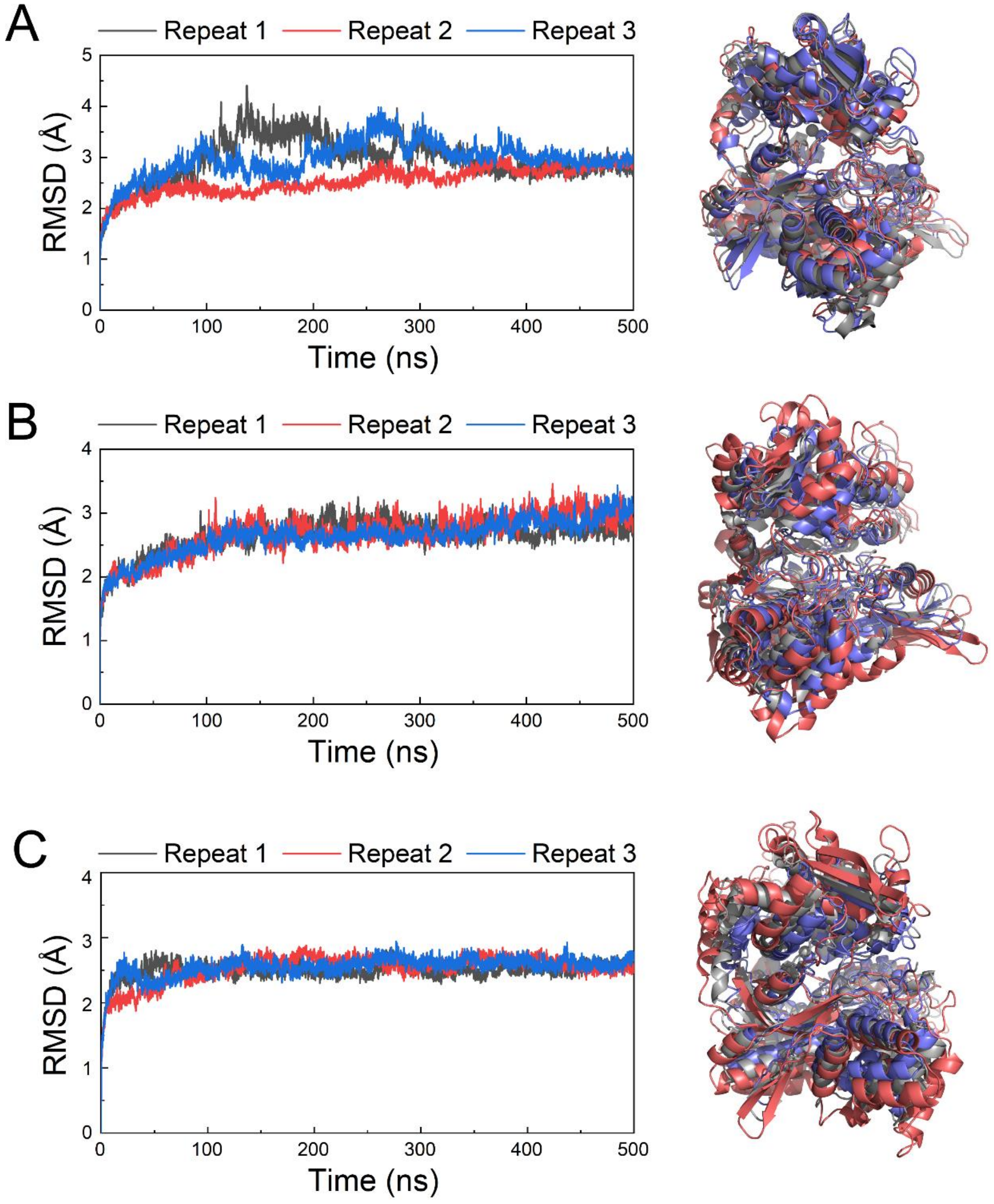

Figure S3. RMSD values and the aligned average structures of three systems during 500 ns MD simulations.

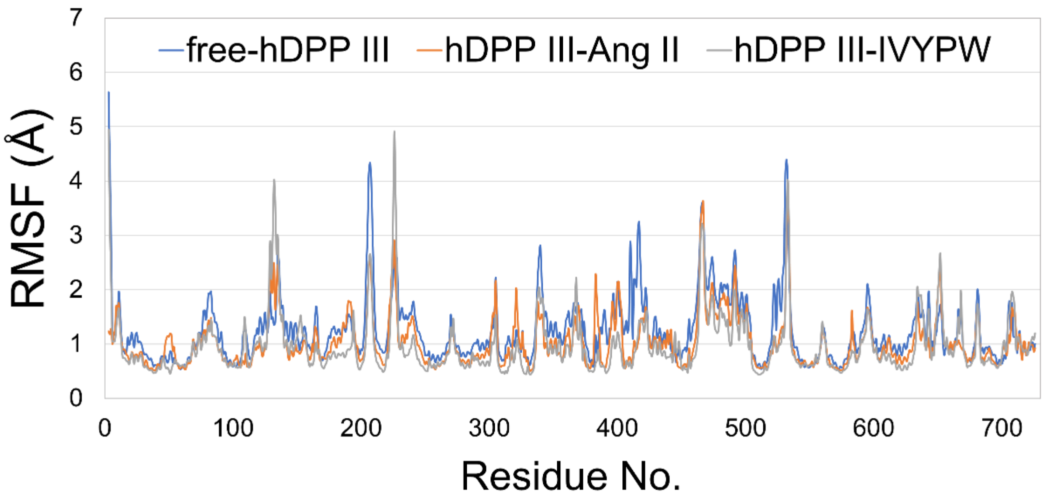

Figure S4. RMSF plots of three systems.

Table S1. Residues contained in each subsite.

Subsite No.	Residues
S2	Glu316, Ile390, Asn391, Ile392, Asn394, Asp396, Arg399, His455, Asp496, Ser504, Glu507, Glu508
S1	Tyr318, Glu329, Phe381, Pro387, Gly389, Ile390, His450, Glu508, His568
S1’	Pro387, Ala388, GLy389, Phe443, Gln446, Val447, His450, Glu512, His568
S2’	Phe109, Tyr318, Pro387, Ala388, Phe443, His568, Arg572
S3’	Ile386, Pro387, Ala388, Val412, Ala416, Phe443, Arg669, Lys670, Ile672

Table S2. The probability of secondary structures of residue Arg421-Lys423 in three repetitions.

		Free-hDPP III		hDPP III-Ang II		hDPP III-IVYPW	
	**Residue**	**α-helix**	**Loop**	**α-helix**	**Loop**	**α-helix**	**Loop**
**Group 1**	**R421**	0.39	0.61	0.81	0.19	0.35	0.47
	**E422**	0.39	0.61	0.81	0.19	0.35	0.45
	**K423**	0.39	0.57	0.81	0.18	0.35	0.62
**Group 2**	**R421**	0.48	0.51	0.71	0.28	0.15	0.65
	**E422**	0.48	0.52	0.71	0.29	0.15	0.85
	**K423**	0.48	0.50	0.71	0.19	0.15	0.62
**Group 3**	**R421**	0.53	0.40	0.67	0.33	0.56	0.43
	**E422**	0.53	0.47	0.67	0.33	0.56	0.44
	**K423**	0.53	0.46	0.67	0.31	0.56	0.43
